# Wisconsin diversity panel phenotypes: spoken descriptions of plants and supporting data

**DOI:** 10.1186/s13104-024-06694-y

**Published:** 2024-01-23

**Authors:** Colleen F. Yanarella, Leila Fattel, Ásrún Ý. Kristmundsdóttir, Miriam D. Lopez, Jode W. Edwards, Darwin A. Campbell, Craig A. Abel, Carolyn J. Lawrence-Dill

**Affiliations:** 1 Iowa State University, 50011 Ames, IA USA; 2 USDA ARS, 50011 Ames, IA USA

**Keywords:** Phenotyping, Maize, Association studies, Audio recordings, Text transcripts, Images, Wisconsin Diversity panel

## Abstract

**Objectives:**

Phenotyping plants in a field environment can involve a variety of methods including the use of automated instruments and labor-intensive manual measurement and scoring. Researchers also collect language-based phenotypic descriptions and use controlled vocabularies and structures such as ontologies to enable computation on descriptive phenotype data, including methods to determine phenotypic similarities. In this study, spoken descriptions of plants were collected and observers were instructed to use their own vocabulary to describe plant features that were present and visible. Further, these plants were measured and scored manually as part of a larger study to investigate whether spoken plant descriptions can be used to recover known biological phenomena.

**Data description:**

Data comprise phenotypic observations of 686 accessions of the maize Wisconsin Diversity panel, and 25 positive control accessions that carry visible, dramatic phenotypes. The data include the list of accessions planted, field layout, data collection procedures, student participants’ (whose personal data are protected for ethical reasons) and volunteers’ observation transcripts, volunteers’ audio data files, terrestrial and aerial images of the plants, Amazon Web Services method selection experimental data, and manually collected phenotypes (e.g., plant height, ear and tassel features, etc.; measurements and scores). Data were collected during the summer of 2021 at Iowa State University’s Agricultural Engineering and Agronomy Research Farms.

## Objective

Formative research using free text descriptions of plant phenotypes along with Natural Language Processing (NLP) methods has demonstrated that computing on plant phenotypes alone can recover known genotype-phenotype associations [1, 2]. Building on these successes, continued efforts to generate plant phenotype descriptions that are both structured (e.g., ontologies) and unstructured (i.e., free text) hold great promise for enabling researchers to advance analytics for phenotypes and traits, especially when these data are made publicly accessible [3].

We developed this dataset as a foundation for analyzing large volumes of spoken phenotype descriptions in a field environment. These phenotype observations were drawn from the Wisconsin Diversity panel, which contains sufficient phenotypic diversity in a field environment for various genotype-to-phenotype analyses [4–6]. Observers generating the datasets were not confined to rigid vocabularies and were not strictly limited to a list of traits to comment on. The protocols that we have developed, along with additional pipeline development, can initiate the use of citizen scientists and become practical for the capture low-cost large-volume free-text phenotype description of plants.

Supplemental to spoken descriptions of plant phenotypes and the text derived from these observations, measurements and scores for traits of interest were also collected as ground truth. Field layout and weather data are reported, along with images of the rows in the field and aerial images from a drone. Consequently, this dataset may be useful to investigators interested in data collected from diversity panels and to those interested in processing natural language and its use in describing scientific phenomena.

The use of this dataset for investigating biological relevance and utility, including developed tools to assist in the use of spoken descriptions for field-based plant phenotype analytics is available [7].

### Data description

This dataset [8] was collected and derived from observations of an experimental field at Iowa State University’s Agricultural Engineering and Agronomy Research Farms in Boone, Iowa. The Wisconsin Diversity panel (686 accessions), an environmental control line (B73, the maize reference line used for genetics and genomics), and 25 positive control accessions were planted in two replicates, and observations were generated over the summer of 2021. This dataset includes the following elements (Table 1).

- Each student participant and volunteer is identified in datasets only by their code names (from the NATO phonetic alphabet).
- Audio text processing data contains the spoken data collected by the volunteers (WAV files) and descriptions of the recordings generated by student participants using Sony ICD-UX570 recorders. Additionally included are metadata (summary statistics) derived from the recordings and code to generate these statistics. Further, all intermediate files (JSON, TXT, and EXCEL files) and code to generate the final cleaned transcripts for all student participant recordings and a subset of the volunteer’s recordings are included. These files provide a resource to investigators to utilize field-collected spoken natural language descriptions of maize plants.
- Methods selection data includes data and code for generating transcriptions using various Amazon Web Services (AWS) Transcribe methods. These methods include using an individualized custom vocabulary for each student participant and an example using data collected by volunteer “Whiskey” as well as a generalized custom vocabulary for each student participant and volunteer Whiskey’s data, and no custom vocabulary. A subset of data was selected to process and compare to a gold standard transcription manually generated to calculate a similarity score to determine the method for transcribing all spoken descriptions collected during the summer of 2021.
- Images of each row in the field were captured with a Canon EOS Rebel T7 camera and Canon EF-S 18-55 mm Image Stabilizer Macro 0.25 m/0.8ft set to 18 mm with AF Stabilizer ON.
- Aerial still images and footage of the experimental field were captured with a Mavic 2 Pro drone by DJI.
- The field data information layout demonstrates the randomizations of the accessions planted and positive controls used. Additionally, seeds planted per row and the Iowa Phytosanitary Corn Field Inspection report conducted on the experimental field are included.
- Field prompting data includes the cards provided to student participants to prompt their behavior while collecting spoken observations in the field, information about the assigned card for each day, and logs for worker data collection. Volunteer field guide cards and logs for data collection are present. Each student participant was instructed to make three complete passes of the field, and volunteers were instructed to make one complete pass of the field. Volunteer “India” completed observations for replicate one only.
- Measurement and scoring data were collected through manual measuring and scoring by trained student participants and volunteers. Plastic measuring sticks with hash marks every 10 cm were used to measure plant height.
- Weather information was collected and reported by The Iowa Environmental Mesonet through Iowa State University [9]. Data for the weather stations nearest the Agricultural Engineering and Agronomy Research Farms for March 2021 to September 2021 and September 2020 to September 2021 are provided.

**Table 1:** Overview of data files/data sets

Label	Name of data file/data set	File types (file extension)	Data repository and identifier (DOI or accession number)
2021 Wisconsin Diversity Panel Dataset	Carolyn_Lawrence_Dill_Maize_WiDiv_Summer_2021_Dataset_June_2023	Directory	CyVerse [8] (10.25739/pvx4-5j31)
	/README.txt	A file with file type:.txt Provides details regarding the subdirectories.	CyVerse [8] (10.25739/pvx4-5j31)
	/audio_text_processing_data	A subdirectory containing file types:.csv,.json,.py,.tar.gz,.txt,.xlsx, and.yaml Demonstrates processing of spoken observations to text files.	CyVerse [8] (10.25739/pvx4-5j31)
	/aws_method_selection	A subdirectory containing file types:.csv,.json,.out,.py,.R,.txt,.wav,.xlsx, and.yaml Demonstrates methods for and accuracy of for transcribing speech-to-text using AWS.	CyVerse [8] (10.25739/pvx4-5j31)
	/camera_images	A subdirectory containing file types:.pptx,.tar.gz,.txt, and.xlsx Contains images of the experimental field rows.	CyVerse [8] (10.25739/pvx4-5j31)
	/drone_images	A subdirectory containing file types:.jpg,.mp4, and.txt Consists of drone-captured still images and videos of the experimental field.	CyVerse [8] (10.25739/pvx4-5j31)
	/field_layout_and_seed_information	A subdirectory containing file types:.pdf,.txt, and.xlsx Includes information about taxa planted and location of taxa in the experimental field.	CyVerse [8] (10.25739/pvx4-5j31)
	/field_prompting_cards_information	A subdirectory containing file types:.png,.psd,.txt, and.xlsx Demonstrates field prompting cards and directions provided to observers.	CyVerse [8] (10.25739/pvx4-5j31)
	/measurement_and_scoring_data	A subdirectory containing file types:.txt and.xlsx Records of measurement and scoring data of the experimental field.	CyVerse [8] (10.25739/pvx4-5j31)
	/weather_data	A subdirectory containing file types:.txt and.xlsx Reports weather data collected from stations in close proximity to the experimental field.	CyVerse [8] (10.25739/pvx4-5j31)

### Limitations

Some audio observations were incomplete due to technical difficulties, including microphone disengagement from the recording devices or observers recording observations for the incorrect row. Also, speech-to-text pipelines and post-process cleaning steps are fallible, leading to transcription inaccuracies. These data were taken over approximately seven weeks, and there were apparent growth and developmental changes throughout the duration of the study. Additionally, the observations within this dataset are for two replicates in the same environment, and additional years, plots, and environments could supplement these available speech data for a more robust dataset.

## Data Availability

The data described in this Data Note can be freely and openly accessed on CyVerse under Digital Object Identifiers (DOI) 10.25739/pvx4-5j31. Please see Table 1 and the references list for details and links to the data.

## Abbreviations

AWS: Amazon Web Services
NLP: Natural Language Processing
